# Ultrasound-Guided Regional Anesthesia: A Narrative Review of Techniques, Safety, and Clinical Applications

**DOI:** 10.7759/cureus.102822

**Published:** 2026-02-02

**Authors:** Fadi A Jamaleddin Ahmad, Joshua A Herrera, Joanna M Saldanha, Akbar Khan, Waleed Nasir, Miriam L Otim, Asjad Y Amin, Nosakhare R Asemota, Sadiq Bhadmus, Farah AlShammari, Aditya Vikkiraman, Izatullah Kamran

**Affiliations:** 1 Family Medicine, American University of the Caribbean School of Medicine, Cupecoy, SXM; 2 Medicine, Universidad Autónoma de Guadalajara, Guadalajara, MEX; 3 Medicine, Royal College of Surgeons in Ireland, Dublin, IRL; 4 Medicine, University Hospitals Bristol and Weston NHS Foundation Trust, Bristol, GBR; 5 Emergency Medicine, Sherwood Forest Hospitals NHS Foundation Trust, Nottinghamshire, GBR; 6 Medicine, Tbilisi State Medical University, Tbilisi, GEO; 7 Medicine, University of Medical Sciences and Technology (UMST), Khartoum, SDN; 8 Medicine, Kursk State Medical University, Kursk, RUS; 9 Medicine, American University of the Caribbean School of Medicine, Cupecoy, SXM; 10 Medicine, Kuwait University, Kuwait City, KWT; 11 Medicine, Pondicherry Institute of Medical Sciences, Puducherry, IND; 12 Medicine, Dow International Medical College, Karachi, PAK

**Keywords:** enhanced recovery after surgery (eras), local anesthetic systemic toxicity (last), lower extremity blocks, needle visualization, opioid-sparing, perioperative analgesia, peripheral nerve blocks, truncal plane blocks, ultrasound-guided regional anesthesia (ugra), upper extremity blocks

## Abstract

Ultrasound-guided regional anesthesia (UGRA) has changed how we manage pain by allowing doctors to visualize nerves and other structures in real time, making the process more accurate, effective, and safe while also reducing risk. This narrative review examines the latest advancements in UGRA techniques, evaluating their efficacy and safety, particularly for peripheral nerve and truncal plane blocks, their potential to diminish opioid consumption, and their clinical applications in perioperative care. This review followed the Scale for the Assessment of Narrative Review Articles (SANRA) quality assessment guidelines and searched six databases for studies published from January 2015 to October 2025, including trials, meta-analyses, cohort studies, reviews, and technical reports. This review aims to give doctors, anesthesia experts, and researchers the newest information and methods about UGRA, focusing on better ways to see needles and use additives like dexamethasone and dexmedetomidine to make regional anesthesia more effective and last longer, which can help lower opioid use and speed up recovery after surgery, while ultrasound guidance makes these techniques safer and more accurate. Another important finding is that real-time imaging and protocols help reduce safety concerns like toxicity, nerve damage, and infection. Artificial intelligence (AI) and robotics in UGRA enhance precision, safety, and recovery, paving the way for further standardization and accessibility.

## Introduction and background

Ultrasound-guided regional anesthesia (UGRA) has revolutionized healthcare, replacing landmark and nerve-stimulation techniques. Real-time visualization of nerves with non-invasive imaging enhances block precision, safety, and success rates. It helps anesthesiologists verify needle placement and assess anesthetic dispersion, improving efficacy and reducing risks such as nerve injury and intravascular injection [1].

Ultrasound guidance has been the global standard in regional anesthesia since 2015. This has led to new techniques and uses. Its consistent outperformance of traditional methods in perioperative pain management is an important development. UGRA offers important clinical advantages over landmark and nerve-stimulator techniques, including safety, accuracy, and effectiveness [2]. Ultrasound allows for the real-time visualization of structures, puncture sites, and the distribution of anesthesia, thereby improving safety and accuracy. As UGRA is wide-ranging and rapidly evolving, covering technical methods, drug use, safety issues, and new technologies, a narrative review approach was chosen to gather current information and provide useful advice for doctors and trainees.

## Review

Methodology

Literature Review, Study Selection, and Inclusion & Exclusion Criteria

This review searched six major databases, including MEDLINE (PubMed), Embase (Ovid), Cochrane Library, Web of Science, Scopus, and Google Scholar, and conducted author screening from August 2025 to October 2025. Table 1 below was adapted to highlight the literature search strategy and methodology sequence. This narrative review aims to provide a general overview of clinical and technical information on UGRA, rather than to conduct a detailed statistical analysis or a systematic review. Also, one seminal background article was added to the introduction, which does not affect the authors' inclusion dates as outlined in Table 1. No formal risk-of-bias assessment or study selection flow diagram was performed.

**Table 1:** Literature search strategy and methodological framework.

Category	Description
Study design	Narrative review
Objective of the search	To summarize current evidence on ultrasound-guided regional anesthesia (UGRA), including techniques, pharmacology, safety, complications, and emerging technologies
Databases searched	MEDLINE (PubMed), Embase (Ovid), Cochrane Library, Web of Science, Google Scholar
Time period covered	January 2015 to October 2025
Search strategy	Database-specific adaptations of structured search strings using MeSH terms and keywords
Key search terms	“Ultrasonography,” “ultrasound-guided,” “regional anesthesia,” “nerve block,” “peripheral nerve block,” “analgesia,” “local anesthetic,” “opioid,” “postoperative pain,” “complications,” “local anesthetic systemic toxicity,” “needle visualization,” “block safety”
Inclusion criteria	Human studies; adult and pediatric populations; clinical trials; observational studies; systematic reviews; meta-analyses; technical reviews; and case reports related to UGRA
Exclusion criteria	Non-English publications; animal or cadaveric studies; abstracts without full text; studies not focused on ultrasound-guided techniques
Rationale for inclusion approach	Broad inclusion is used to capture the multidisciplinary and evolving nature of UGRA across clinical practice
Study selection process	Titles and abstracts screened by authors; relevant full texts reviewed for inclusion
Nature of review	Narrative review (not a systematic review or meta-analysis)
Rationale for narrative design	Heterogeneity in study design, outcomes, and evolving technologies precluded quantitative synthesis; the narrative approach allowed clinical integration
Methodological limitations	Potential publication bias, exclusion of non-English studies, lack of formal risk-of-bias assessment, and variability among included studies

Quality Assessment

Each author independently screened the assigned studies, with discrepancies resolved through discussion in accordance with the Scale for the Assessment of Narrative Review Articles (SANRA) quality assessment standards, as explained in Table 2.

**Table 2:** SANRA evaluation of the narrative review. UGRA: ultrasound-guided regional anesthesia; SANRA: Scale for the Assessment of Narrative Review Articles.

Criterion	Score (0–2)	Brief justification
Justification of importance	2	Clearly establishes the clinical relevance and timeline of UGRA innovations.
Statement of aims	2	Objectives clearly defined: Evidence, methodology, and uses of UGRA (2015-2025).
Literature search	1	Six databases, transparent inclusion/exclusion criteria.
Referencing	2	139 current and relevant sources up to 2025, correctly formatted.
Scientific reasoning	1.5	Logical synthesis but lacks bias assessment or quantitative pooling.
Presentation of data	1.5	Well-structured with tables/figures; lacks formal grading of evidence.
Total score	10/12	Excellent-quality narrative review with minor methodological limitations.

Objective of the Review

This narrative review highlights the clinical applications, safety, and technical aspects of UGRA. It will cover commonly used techniques and agents in UGRA, as well as measures to reduce complications. In addition, it will discuss innovations in UGRA that may further enhance its safety and efficacy.

Fundamentals of ultrasound for regional anesthesia

Equipment & Probes: Image Optimization

Ultrasound-guided local anesthesia allows real-time needle placement and reduces complications like insufficient blockade or toxicity. Table 3 lists various types of clinical ultrasound probes, each with distinct configurations and frequencies [3].

**Table 3:** Comparison of ultrasound probe types based on their shape, depth, and clinical applications. Data compiled by the authors from reference [3].

Probes	Shape	Depth	Application
Linear array	Rectangular-shaped & flat	High frequency, ranging from 3 MHz to 19 MHz & penetrates to a depth of up to 10 cm	Visualize superficial structures & help with the administration of superficial regional anesthesia
Phased array	Square-shaped & small	Low frequency, ranging from 1 to 5 MHz	Visualize a single point (e.g., ribs) by directing the beams to a single point in proximity
Curvilinear array	Curved & not flat	Low frequency, ranging from 1 to 5 MHz, and penetrates up to 30cm in depth	Visualize deep structures (e.g., transabdominal ultrasounds)

Needle Visualization Techniques

**Figure 1:**
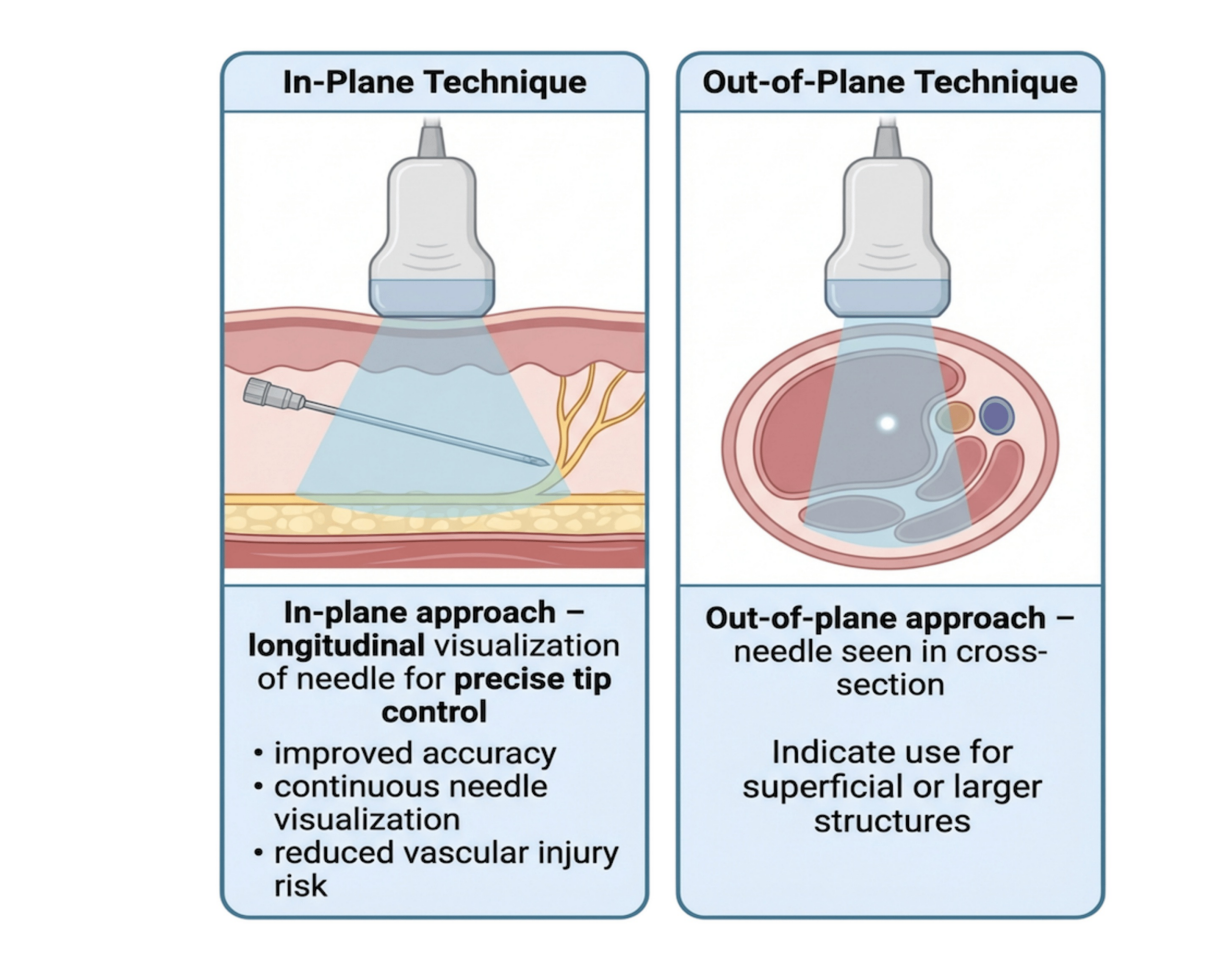
Ultrasound-guided needle visualization techniques: in-plane and out-of-plane approaches. This illustration compares the in-plane and out-of-plane ultrasound-guided needle approaches. The in-plane technique allows longitudinal visualization of the needle shaft and tip, facilitating precise needle control and improved safety. The out-of-plane technique visualizes the needle in cross-section and is commonly used for superficial or larger anatomical targets. The selection of technique depends on anatomical location, depth, and procedural requirements. Image credit: Created by the authors using BioRender (scientific illustration software).

Two primary visualization methods are in-plane and out-of-plane, as shown in Figure 1. In-plane offers a longitudinal view for accuracy, while out-of-plane positions the probe perpendicular to the needle. Probe choice and depth affect visibility. Out-of-plane suits superficial or large structures, but in-plane is preferred for precise needle-tip control to avoid vessel damage [4].

Asepsis, Ergonomics, and Probe Handling

To prevent infections from the ultrasound probe or gel, use a disposable sleeve or alternatives such as surgical gowns, gloves, or non-lubricated condoms, which are cost-effective and accessible. The sterile cover should extend from the probe to the cable to prevent contamination. It should be sterilized after each use with a high-level disinfectant, such as ortho-phthalaldehyde, hydrogen peroxide, glutaraldehyde, or peracetic acid [5]. The anesthetist should maintain proper posture for comfort, correctly position the patient for the nerve block, and adhere to aseptic protocols to ensure patient safety. The probe is manipulated through five maneuvers: sliding, tilting, rotating, rocking, and applying pressure to improve nerve and needle visibility [6].

Pharmacology & peri-block management

Local Anesthetics: Agents, Concentration/Volume Strategies, and Toxicity Thresholds

In UGRA, selecting the appropriate local anesthetic and dose is crucial for both safety and effectiveness. Common amide anesthetics are ropivacaine, bupivacaine, and levobupivacaine, which are preferred because they last longer and are less likely to cause harm. The concentration and volume vary by block type. Hydrodissection with saline before injecting local anesthetic has been shown to accelerate the onset and spread of infraclavicular blocks [7]. Toxicity levels can vary among individuals, but it is important to stick to the maximum doses and inject slowly while checking [8]. Table 4 lists common anesthetic agents, their recommended doses for adults and children, and any reported side effects [9-12].

**Table 4:** Local analgesics are divided by block type, dosage, onset, and toxicity. Data compiled by the authors from the following references: ropivacaine [9]; levobupivacaine [10]; ropivacaine [11]; bupivacaine [12]. US: ultrasound; TAP: transversus abdominis plane; ED_50_: dose of drug that provides effects in 50% of the population. ED_95_: dose of medication that produces 95% of its maximal effect in a specific population. T_max_: time taken for a drug to reach its maximum plasma concentration.

Agent	Block Type	Recommended dosage	Onset	Effects of toxicity
Ropivacaine	US-guided median nerve block (trigger thumb)	ED₅₀: 0.9 mL (1–3 years), 1.4 mL (3–6 years); ED₉₅: ~1.5–1.7 mL (0.2%)	Minutes	No adverse events reported at these doses
Levobupivacaine	US-guided TAP block	0.2% at 0.2 mL/kg or 0.1% at 0.4 mL/kg (~0.4 mg/kg), adults only	Tmax ~17 min	Median peak plasma ~0.315 µg/mL; no toxicity observed
Ropivacaine	US-guided TAP block (lower abdominal surgery)	~150–200 mg total dose (adult; weight-dependent; ≤3 mg/kg)	Peak plasma ~30 min	Mean peak plasma ~2.54 µg/mL; unbound ~0.14 µg/mL; no clinical toxicity
Bupivacaine	US-guided axillary brachial plexus block	0.25–0.375% bupivacaine ± epinephrine; ~3–5 mL per nerve (adults)	~10–20 min; duration ~4–12 h	Serum levels may approach toxic thresholds with high-volume dosing; adherence to weight-based dosing reduces risk

Adjuvants (Dexamethasone, Dexmedetomidine, Clonidine, Epinephrine): Evidence & Controversies

Adjuvants extend block duration and enhance analgesia, but their efficacy and safety differ. Table 5 lists common UGRA adjuvants with their pros and cons [13-19].

**Table 5:** Commonly used adjuncts in UGRA with their advantages and drawbacks. Data compiled by the authors from the following references: dexamethasone [13-16]; sexmedetomidine [17,18]; clonidine [18]; epinephrine [13,19]. UGRA: ultrasound-guided regional anesthesia.

Adjuvant	Dose/route	Effect on block duration	Mechanism & benefit	Risks
Dexamethasone	4–10 mg, perineural or IV	Prolongs by 4–8 hours	Anti-inflammatory, reduces nociceptive transmission	Neurotoxicity debated; perineural vs. IV efficacy similar in some studies
Dexmedetomidine	0.5–1 µg/kg perineural	Prolongs duration, faster onset	α2-agonist causing vasoconstriction and sedation	Bradycardia, sedation, hypotension
Clonidine	~1–2 µg/kg or fixed dose	Moderate prolongation	α2-agonist	Sedation, hypotension; less potent than dexmedetomidine
Epinephrine	5 μg/mL	Slight prolongation	Vasoconstriction, a marker for intravascular injection	Possible ischemia in end-arterial areas

Dexamethasone is the most effective adjunct. Studies show that dexamethasone helps maintain pain relief longer with erector spinae and serratus anterior plane blocks, leading to better results. Adding dexmedetomidine to ropivacaine for an erector spinae plane block makes pain relief and recovery better, but it also raises the risk of sedation and bradycardia [17]. Clonidine, once common, has been replaced mainly by dexmedetomidine due to its weaker analgesic effects and less favorable side effect profile [18]. Epinephrine primarily narrows blood vessels, which slows its absorption and indicates that it was injected into a blood vessel; it can also extend the duration of analgesia.

Single-Shot vs. Continuous Catheters: Indications, Pump Settings, and Troubleshooting

Single-shot blocks provide reliable pain relief for short- to medium-duration procedures and are widely used for their simplicity [20]. Continuous catheter techniques provide extended analgesia, especially in shoulder and thoracic surgery, allowing the titration of local anesthetics. Infusions typically use low-concentration ropivacaine (0.1-0.2%) at 4-8 mL/hr, allowing patient-controlled boluses [21]. Programmed intermittent bolus (PIB) regimens may improve spread and analgesic quality, though the evidence remains evolving [15]. Catheter complications include dislodgement, infection, anesthetic toxicity, and technical difficulties; careful monitoring is essential.

Monitoring, Sedation, and Documentation Standards

Safe UGRA requires appropriate patient monitoring, including an electrocardiogram (ECG), pulse oximetry, and blood pressure monitoring. To keep the patient able to talk to the doctor, sedation should be kept to a minimum so that nerve irritation or systemic toxicity can be found early. Keeping a close watch on the patient after the block is crucial for spotting signs of local anesthetic systemic toxicity (LAST), with observation time based on the block's risk, and for quickly addressing any problems that arise. Documentation must capture block details, drug doses, onset time, adverse events, and analgesic outcomes to guide clinical care and quality assurance. In the context of continuous catheters, regular assessment of the catheter site and infusion parameters is essential for patient safety [21].

Safety, complications, and risk reduction

LAST: Prevention, Recognition, and Lipid Rescue Algorithm

Prevention of LAST: UGRA reduces the amount of local anesthetic needed, lowering the risk of toxicity by visualizing the needle tip and anesthetic spread [22,23]. Intravascular injection is common and causes severe LAST; real-time visualization is crucial to prevent it. Using levobupivacaine or ropivacaine, which have better cardiac safety and higher toxicity thresholds than bupivacaine, is a vital risk-reduction measure [24].

Procedural vigilance involves gentle, frequent aspiration during injection (not always reliable), administering small doses (3-5 mL) of local anesthetic with pauses to monitor the patient, and using an intravascular marker like epinephrine (1:200,000 or 5 mcg/mL) for early warning through increased heart rate [25,26]. Nerve stimulation as an adjunct provides safety; a sudden loss of motor response after a test dose can indicate intravascular placement [26].

Recognition of LAST: Early recognition of LAST is crucial for prompt treatment and preventing irreversible cardiovascular collapse. Clinicians must be vigilant for subtle prodromal signs, particularly neurological ones, such as metallic taste, perioral numbness, tinnitus, lightheadedness, or visual disturbance [27]. High suspicion is crucial during or after a local anesthetic injection. Talking with an awake patient is the best way to monitor for early detection.

Lipid rescue algorithm: The way we treat LAST has changed significantly with the use of intravenous lipid emulsion (ILE) therapy, which helps remove lipid-soluble local anesthetics from the blood and heart tissue. The American Society of Regional Anesthesia and Pain Medicine (ASRA) has formulated a definitive algorithm that must be readily accessible wherever regional blocks are administered and in cases of suspected LAST [28], as presented in Table 6.

**Table 6:** Management algorithm for local anesthetic systemic toxicity (LAST), outlining stepwise interventions from discontinuation of anesthetic administration to post-stabilization intensive monitoring. Data compiled by the authors from reference [28]. ILE: intravenous lipid emulsion; ACLS: advanced cardiac life support.

Step	Action/Recommendation	Details/Notes
1. Stop injection	Discontinue the local anesthetic immediately	Request assistance and prepare for emergency management
2. Airway & oxygenation	Prioritize airway management and provide supplemental oxygen	Prevent hypoxia and acidosis, which exacerbate LAST
3. Seizure control	Administer benzodiazepines or propofol	Use propofol only if cardiovascularly stable
4. Lipid emulsion therapy (ILE)	Initiate 20% lipid emulsion	Bolus: 1.5 mL/kg over 2–3 min. Infusion: 0.25 mL/kg/min & repeat as needed; continue for ≥10 min after hemodynamic stabilization
5. Avoid substitutes	Do not use propofol in place of ILE	Propofol has insufficient lipid content and a higher risk of adverse effects
6. Cardiovascular support (Modified ACLS)	Manage cardiovascular collapse per advanced cardiac life support (ACLS) with modifications	Avoid: vasopressin, calcium channel blockers, beta-blockers. Use low-dose epinephrine (<1 mcg/kg) to minimize worsening LAST
7. Post-stabilization monitoring	Transfer to ICU for prolonged observation	Monitor for rebound toxicity as lipid emulsion is metabolized

Nerve Injury Risk, Intraneural Injection, and Monitoring Injection Pressure

Preventive efforts can lead to improved outcomes, lower post-procedure complications, and increased patient satisfaction. Table 7 outlines key aspects to consider during needle insertion and summarizes the key points for each step.

**Table 7:** Summary of nerve injury risk, intraneural injection, and injection pressure monitoring in UGRA. Data compiled by the authors from the following references: nerve injury risk [29]; patient-related risk factors [30]; intraneural injection [31]; injection pressure monitoring [32]; preventive strategies [31]. UGRA: ultrasound-guided regional anesthesia.

Aspect	Summary	Key Points/Evidence
Nerve injury risk	Permanent neurological injury after UGRA is rare, reported at a 0.1–0.4% incidence. Risk increases with pre-existing neuropathies, advanced age, or connective tissue disorders.	Proper pre-procedural neurological exam and ultrasound-guided needle control are essential to reduce direct nerve trauma.
Patient-related risk factors	Conditions like diabetic or chemotherapy-induced neuropathy and Ehlers–Danlos syndrome heighten vulnerability to nerve injury.	Careful screening and documentation before the block are advised.
Intraneural injection	Previously avoided, but recent studies show low-pressure, subepineural injections may be safe under expert supervision.	Controlled intraneural techniques can enhance block efficacy but require awake or lightly sedated patients to detect paresthesia.
Injection pressure monitoring	High injection pressure (>15 psi) indicates needle misplacement (in fascicle, tendon, or vessel). Real-time pressure monitoring prevents intraneural injury.	Combining ultrasound visualization with pressure manometry forms a dual-safety approach.
Preventive strategies	Stop injection immediately if high resistance or pain occurs; reassess under ultrasound before resuming.	Reduces risk of neuropraxia and ischemic injury during peripheral nerve blocks.

Vascular Puncture/Hematoma: Anticoagulation/Antiplatelet Considerations

Vascular puncture can be prevented by careful pre-procedural scanning and needle visualization. A thorough B-mode and color Doppler ultrasound should identify the target nerve and map nearby arteries and veins before needle insertion. Keeping the needle tip visible, preferably in-plane, is vital throughout. Hydrodissection can also help move vessels out of the needle path [7]. When aspirating blood, retract the needle and press for 5-10 minutes, then observe for an additional five minutes; this method is suitable for superficial blocks.

The management of patients on antithrombotic medications requiring regional anesthesia is complex. It must be guided by the latest societal recommendations, primarily from the ASRA and the European Society of Anaesthesiology and Intensive Care (ESAIC). The most recent guidelines emphasize a patient-specific, multidisciplinary risk-benefit analysis [33]. The key point of the guidelines is stratifying risk by block site. Superficial blocks, such as femoral, popliteal, sciatic, and transversus abdominis plane blocks, carry a much lower risk of serious [34].

Infection Prevention for Single-Shot and Catheter Techniques

Single-shot nerve blocks aim for surgical asepsis, which means the person performing the procedure must wash their hands for two minutes, wear sterile gloves, use a single-use ultrasound cover, and apply sterile gel. Continuous peripheral nerve blocks carry a higher risk of infection because they involve indwelling foreign objects. Strict aseptic standards, often with a full-body drape, are essential during insertion. A study comparing catheter-over-needle and catheter-through-needle techniques found that infection rates were the same for both. However, one design showed greater dislodgement, which can indirectly increase the risk of infection if reinsertion or site complications occur, thereby increasing the risk of contamination [35]. Post-insertion catheter care involves using sterile, transparent dressings to allow daily site checks for signs of inflammation, such as erythema, edema, or purulence. There is no proof that using peripheral nerve catheters with routine prophylactic antibiotics is advantageous. If signs of infection (local signs, fever, elevated WBC count) occur, remove the catheter, culture the tip, and initiate appropriate antibiotics. Studies on continuous blocks, like erector spinae and paravertebral catheters, show low infection rates when strict protocols are followed [36].

Pneumothorax, Diaphragmatic Paresis, and Urinary Retention Risks

Pneumothorax commonly occurs with blocks near the lung apex, like supraclavicular and paravertebral blocks. Its incidence has decreased with the use of ultrasound, enabling operators to identify the pleural line and avoid needle contact [37]. Symptoms may be immediate (such as sharp pain or cough) or delayed (including dyspnea or tachycardia). Diagnosis requires suspicion and is confirmed with a chest X-ray or ultrasound.

Hemidiaphragmatic paralysis is a common result, not a problem, of the interscalene brachial plexus block, which happens because of phrenic nerve involvement. It occurs in nearly all patients with standard volumes (>15 mL), making the block relatively contraindicated in those with severe respiratory issues who cannot tolerate approximately 25% decrease in lung capacity. Recent studies focus on mitigation strategies [38].

Urinary retention is associated with neuraxial anesthesia (spinal, epidural) due to the S2-S4 nerve block, which affects bladder function. It is rare after peripheral nerve blocks, but it can happen with extensive bilateral lower limb blocks or risk factors like benign prostatic hyperplasia. Falls are a risk after femoral or sciatic nerve blocks, particularly in outpatient surgery, due to the resulting motor weakness. Patients need education on weight-bearing, knee brace use, and clear instructions until the motor block subsides. Knowing the anatomy of each block helps prevent and manage these effects [35,39].

Quality Assurance, Incident Reporting, and Credentialing

A quality assurance program actively monitors and improves a regional anesthesia service using key performance indicators (KPIs), such as block success rates, complication rates, and patient satisfaction. These KPIs include surgical anesthesia, postoperative analgesia, LAST rate, nerve injury, infection, and block failures [29]. Periodic morbidity and mortality reviews, as well as departmental audits, review complex cases and complications in a non-punitive, educational setting, which are invaluable for collective learning and system improvement [40].

A key reactive element of a safety culture is voluntary, non-punitive incident reporting, including near-miss incidents that could have caused harm but did not due to chance or intervention. These reports reveal hidden flaws in equipment, communication, or protocols, often underlying safety issues [29].

Credentialing and privileging should ensure that clinicians performing UGRA possess the necessary skills and competencies. An integrated credentialing career map must include several steps: didactic education, which provides a basic understanding of ultrasound physics, sonic anatomy, pharmacology, and complication management; simulation-based training, which involves practical training in a non-hazardous setting with phantoms and high-fidelity simulators to develop needle skills, scan interpretation, and the treatment of rare emergencies, such as LAST; and proctored practice, in which a set number of critical blocks must be conducted with the direct oversight of a competent, experienced mentor with documented competence through various complexities [29].

Upper extremity blocks (brachial plexus & distal)

**Figure 2:**
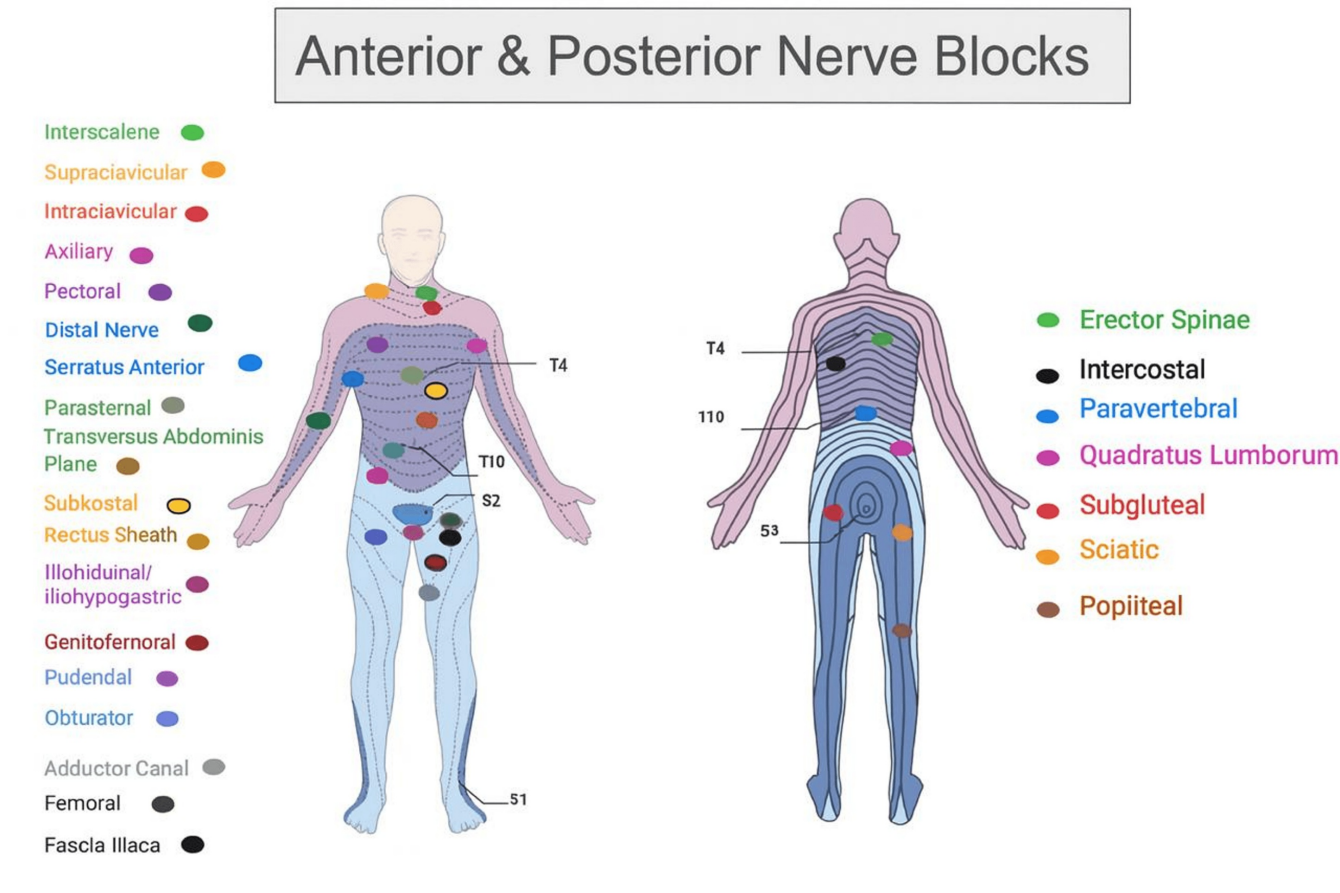
Landmark for the anterior and posterior nerve blocks. Image credit: Created by the authors using BioRender (scientific illustration software).

Upper-extremity blocks are highlighted in Table 8 [38-48], which illustrates the types of regional blocks and their visual landmarks (Figure 2). Descriptions and indications, technique/key anatomy, and advantages/considerations for performing UGRA per block are also provided.

**Table 8:** Summary of upper extremity blocks. Data compiled by the authors from the following references: interscalene block (ISB) [38,39,41]; supraclavicular block (SCB) [42-44]; infraclavicular block (ICB) [43,45,46]; axillary [47]; distal nerve blocks (median, ulnar, radial, musculocutaneous) [48]. COPD: chronic obstructive pulmonary disease.

Block	Description/Indications	Technique/Key Anatomy	Advantages/Considerations
Interscalene block (ISB)	Standard for postoperative analgesia after shoulder surgeries; targets superior trunk (C5–C6) of brachial plexus	Ultrasound-guided (UG) identification of anterior/middle scalene muscles and C5–C7 roots ("traffic light" sign); needle in-plane lateral-to-medial toward C5–C6 sheath; low volume (5–10 mL) reduces risk of hemidiaphragmatic paresis (HDP)	Excellent shoulder analgesia; risk of HDP, but ipsilateral extra-fascial injection reduces phrenic nerve involvement
Supraclavicular block (SCB)	“Spinal of the arm”; dense anesthesia for the upper limb (mid-humerus and below); ideal for trauma and emergency surgery	UG at supraclavicular fossa; use the first rib as a safety backstop; needle advanced lateral-to-medial toward plexus; subclavian artery and pleura as landmarks	Fast onset, comprehensive anesthesia; less phrenic nerve risk than ISB; pleural puncture avoided with correct technique; low volumes reduce HDP
Infraclavicular block (ICB)	For surgeries below the elbow; suitable for catheter placement for prolonged analgesia	Targets the brachial plexus cords around the axillary artery under the pectoral muscles; a single injection behind the artery or the costoclavicular approach for tighter cord packing	Long-lasting analgesia; low pneumothorax/phrenic risk; ideal for COPD/obese patients; continuous catheters stabilize well; costoclavicular variant provides faster onset
Axillary block	Distal brachial plexus block for forearm, wrist, and hand surgeries; ideal for outpatients and those with respiratory disease	Inject around the axillary artery to block the median, ulnar, radial, and musculocutaneous nerves; ultrasound-guided multiple injections improve efficacy	Safe (away from pleura/phrenic nerve); minimal respiratory risk; excellent for ambulatory settings; multiple injections yield better success
Distal nerve blocks (median, ulnar, radial, musculocutaneous)	Target individual nerves at the elbow/wrist for selective sensory anesthesia and motor preservation	UG injections (3–5 mL per nerve); typically used for precision procedures or rescue blocks	Motor-sparing; low anesthetic volume minimizes toxicity; superior to IV regional anesthesia for carpal tunnel; highly safe and targeted

Thoracic & truncal blocks

Thoracic and truncal blocks are outlined in Table 9 [49-56], showing the types of regional blocks. Their visual landmarks are displayed in Figure 2, along with descriptions, indications, techniques/key anatomy, and advantages or considerations for performing UGRA with each block.

**Table 9:** Summary of thoracic and truncal nerve blocks. Data compiled by the authors from the following references: paravertebral block (PVB) [49-51]; erector spinae plane (ESP) block [52]; pectoral nerve blocks (PECS I/II) [53]; serratus anterior plane block (SA) [54]; intercostal nerve block (ICB) [55]; parasternal & interfascial plane blocks (PIFB, TTP) [56]. TTP: transversus thoracic muscle plane block; LA: local anesthetic.

Block	Description/Indications	Technique/Key anatomy	Advantages/Considerations
Paravertebral block (PVB)	Effective postoperative analgesia for thoracic & breast surgeries; blocks sympathetic & somatosensory signals for unilateral chest/abdominal pain	Inject local anesthetic near spinal nerves; the needle is advanced straight (no tilt) and redirected caudally to avoid pleural puncture	Safer than an epidural (less hemodynamic instability, nausea, urinary retention)
Erector spinae plane (ESP) block	For thoracic & abdominal somatic and visceral pain; also effective for acute and chronic cancer, acute pain	Inject local anesthetic into the fascia of the erector spinae near the transverse process (ultrasound-guided); spreads cephalocaudally to paravertebral/epidural/intercostal spaces	Simple, versatile, low complication rate; ~3.4 mL per dermatome
Pectoral nerve blocks (PECS I/II)	Analgesia for chest wall surgeries (e.g., mastectomy, axillary dissection, and port placement)	PECS I: Inject between pectoralis minor & major at the 3rd rib; PECS II: Between pectoralis major & serratus anterior	Blocks lateral & medial pectoral nerves; effective and easy with ultrasound
Serratus anterior plane block (SA)	For trauma (e.g., rib fractures) and thoracic wall pain	Inject LA between latissimus dorsi & serratus anterior, or beneath SA above intercostal muscles; identify thoracodorsal artery via Doppler	Blocks intercostobrachial, lateral cutaneous (T3–T9), long thoracic & thoracodorsal nerves
Intercostal nerve block (ICB)	Pain relief post-thoracic surgery & chest trauma	LA is injected into the intercostal space outside the parietal pleura at appropriate levels	Reduces respiratory complications; risk of systemic absorption causing hypotension/CNS effects; lasts 6–8 hours
Parasternal & interfascial plane blocks (PIFB, TTP)	Regional analgesia for chest wall pain (surgery, trauma, respiratory distress)	LA injected between muscles, not targeting anterior cutaneous branches; includes parasternal intercostal plane block	Good alternative to neuraxial blocks (paravertebral, epidural, spinal), especially in cardiac surgery or anticoagulated patients

Abdominal wall & pelvic blocks

Abdominal and pelvic blocks are summarized in Table 10 [57-68], which lists the types of regional blocks and their corresponding visual landmarks (Figure 2). The descriptions, indications, technique, key anatomy, and advantages or considerations for performing UGRA per block are also provided.

**Table 10:** Summary of abdominal wall & pelvic blocks. Data compiled by the authors from the following references: transversus abdominis plane (TAP) blocks (subcostal, lateral, posterior) [57,58]; rectus sheath block (RSB) [59,60]; quadratus lumborum (QL) blocks (QL1, QL2, transmuscular) [61-64]; ilioinguinal/iliohypogastric, genitofemoral & pudendal blocks [13,19,65-67]; obturator nerve block (ONB) (anterior & posterior approaches) [68]. LA: local anesthetic; PNB: pudendal nerve block; ONB: obturator nerve block.

Block	Description/Indications	Technique/Key anatomy	Advantages/Considerations
Transversus abdominis plane (TAP) blocks (subcostal, lateral, posterior)	Used for abdominal surgeries (laparoscopic cholecystectomy, hysterectomy, varicocelectomy, nephrectomy); reduces postoperative pain, opioid use, and nausea	Injection between internal oblique and transversus abdominis; variants: subcostal (upper abdomen), lateral (flank), posterior (near lumbar triangle)	Improves pain control, ambulation, and sleep; subcostal and combined approaches are superior to single TAP
Rectus sheath (RS) block	Used in hernia, abdominal, and prostate surgeries	LA injection into the posterior rectus sheath under ultrasound guidance, bilateral for midline incisions	Reduces pain, opioid use, nausea, and vomiting postoperatively; effective for both open and laparoscopic procedures
Quadratus lumborum (QL) blocks (QL1, QL2, transmuscular)	Used in abdominal, hip, and renal surgeries; also used for chronic pain relief	QL1: lateral (superficial to QL). QL2: posterior (between QL & thoracolumbar fascia). QL transmuscular: between QL & psoas major; ultrasound-guided	Provides visceral and somatic analgesia; long duration; effective for chronic and postoperative pain; combination with fascia iliaca block enhances recovery; comparable to epidural in pediatrics
Ilioinguinal/iliohypogastric (II/IH), genitofemoral (GF) & pudendal (PN) blocks	Used in unilateral hernia repair, prostatectomy, hemorrhoid repair, and urethroplasty; also for obstetric analgesia	II/IH: between the internal oblique and the transversus abdominis. Triple block: includes II/IH + GF + PNB: near ischial spine or transperineal; all under ultrasound guidance	II/IH is more effective than wound infiltration; triple block is superior to spinal alone; PNB improves pain scores vs. caudal and local infiltration and reduces opioid use and recovery time
Obturator nerve (ON) block (anterior & posterior approaches)	Prevents adductor jerk during transurethral resection of bladder tumor (TURBT); treats hip/knee pain and spasticity	Anterior: between adductor brevis & longus. Posterior: between adductor brevis & magnus; ultrasound or nerve stimulator guided	Using spinal and ONB nerve stimulators enhances surgical safety in TURBT, reduces spasm and pain, and provides safe and precise ultrasound-guided techniques for reflex inhibition.

Lower extremity blocks

Lower-extremity blocks are highlighted in Table 11 [69-82], which illustrates the types of regional blocks and their visual landmarks, as shown in Figure 2. Descriptions and indications, technique/key anatomy, and advantages/considerations for performing UGRA per block are also provided.

**Table 11:** Summary of lower extremity blocks. Data compiled by the authors from the following references: femoral & fascia iliaca compartment blocks (emphasizing the suprainguinal approach) [69-72]; adductor canal (saphenous) block [73-75]; sciatic nerve & ankle blocks (subgluteal, popliteal) [76-79]; IPACK & posterior knee analgesia strategies [80-82]. IPACK: infiltration between the popliteal artery and the capsule of the knee.

Block	Description/Indications	Technique/Key anatomy	Advantages/Considerations
Femoral & fascia iliaca compartment blocks (emphasizing the suprainguinal approach)	Used for femoral neck fractures, hip arthroplasty, and revision surgeries; provides pre- and postoperative analgesia	Ultrasound-guided injection into the fascia iliaca compartment (suprainguinal approach improves spread to femoral and lateral femoral cutaneous nerves); often combined with spinal anesthesia	Effective analgesia, reduced opioid use, safe for elderly trauma patients; suprainguinal technique enhances block coverage, and may be an alternative to spinal morphine (needs further study)
Adductor canal (saphenous) block	Provides analgesia for knee and medial leg procedures such as total knee arthroplasty (TKA)	Ultrasound-guided injection into the adductor canal near the femoral artery under the sartorius blocks the saphenous nerve	Preserves quadriceps motor function; safer (low vascular puncture risk); excellent for early ambulation post-TKA
Sciatic nerve & ankle blocks (subgluteal, popliteal)	Analgesia for foot, ankle, and posterior leg surgeries; useful in pediatric patients for compliance and pain control	Ultrasound-guided at the subgluteal or popliteal level; for ankle block, targets tibial, deep/superficial peroneal, sural, and saphenous nerves	Effective perioperative analgesia: heating ropivacaine (30°C) accelerates onset and improves quality; duration similar to ankle block; enhances efficiency without added risk
IPACK & posterior knee analgesia strategies	Pain control after total knee arthroplasty (TKA); complements the adductor canal and femoral blocks	Injection between the popliteal artery and the posterior knee capsule (ultrasound-guided); ~20 mL ropivacaine is sufficient	Reduces opioid use; preserves motor function of tibial and peroneal nerves; comparable efficacy to suprainguinal fascia iliaca block; safe addition to multimodal analgesia

Cost-effectiveness, workflow, and role within Enhanced Recovery After Surgery (ERAS) pathways across specialties

While UGRA may require additional equipment, training, and higher costs, the improved postoperative efficiency offsets these long-term expenses. Despite higher initial costs, UGRA enhances post-anesthesia care unit (PACU) throughput, reduces length of stay (LOS), and minimizes complications, resulting in broader cost benefits. A retrospective study found that ERAS protocols decreased hospital stay from 3.0 to 2.1 days (p < 0.0001) [83].

The combination of point-of-care ultrasound (POCUS) with techniques like focused assessment with sonography for trauma (FAST) has improved anesthesiology by making it possible to assess the airway, heart function, and trauma accurately. While successful outcomes depend on training, rapid detection of injury supports intervention, making POCUS essential in perioperative management [84]. These workflow and cost improvements align with ERAS protocols, which involve multiple disciplines and evidence-based approaches to optimize outcomes from preoperative to postoperative care. As shown in Table 12 [83-86], combining UGRA and POCUS with ERAS helps maintain stable clinical outcomes, improves hospital efficiency, and saves money in the long run.

**Table 12:** Steps to achieving optimal clinical effectiveness and outcomes. Data compiled by the authors from the following references: preoperative planning & assessment [83]; ultrasound-guided regional anesthesia (UGRA) [84]; intraoperative management [85]; postoperative phase (PACU) [83]; ERAS pathway integration [85-86]; clinical outcome [86]. PACU: post-anesthesia care unit; ERAS: Enhanced Recovery After Surgery; POCUS: point-of-care ultrasound; PONV: postoperative nausea and vomiting.

Step	Phase/Title	Key points
1	Preoperative planning & assessment	Evaluate the patient’s injuries, surgical type, and suitability for regional anesthesia, and educate the patient on ERAS expectations and pain management plan
2	Ultrasound-guided regional anesthesia (UGRA)	Identify the appropriate block for the surgical site, and prepare the POCUS setup for airway and vascular guidance
3	Intraoperative management	Administer multimodal analgesia (regional + non-opioid systemic), and minimize intraoperative opioid dosing
4	Postoperative phase (PACU)	Monitor block effectiveness and pain control, and observe for complications (PONV, respiratory depression, and hypotension)
5	ERAS pathway integration	Implement early oral intake and mobilization, continue multimodal analgesia regimen, and encourage physiotherapy involvement (orthopedic cases)
6	Clinical outcome	Reduced length of stay via faster recovery, minimized opioid use and side effects, improved sleep, comfort, and satisfaction, and lower postoperative complication rates

Emerging technologies

Artificial Intelligence (AI)-Assisted Image Recognition and Probe Guidance

AI aids image recognition and improves nerve block teaching. A trial showed AI significantly reduced paresthesia (11 (4.12%) vs. 36 (14.06%), P = 0.000093) and injection pain (2.25% vs. 6.64%, P = 0.025). AI reduces nerve injury and boosts trainee confidence [87]. AI is an emerging topic with the potential to improve accuracy and decrease side effects.

Needle-Tracking Enhancements and Pressure-Sensing Injectables

Adding features like needle-tracking enhancers, pressure-sensing injectables, and UGRA can improve needle placement and tracking. A study showed that the catheter-over-needle (CON) method was more effective than the catheter-through-needle (CTN) method in ultrasound-guided femoral nerve blocks, reducing procedure time and leakage [88].

Robotics

UGRA could influence robotics by enabling minimally invasive procedures with fewer side effects and shorter hospital stays. One study indicated that bilateral low deep serratus anterior plane (DSAP) blocks alleviate postsurgical pain and cut opioid use after trans-subxiphoid robotic thymectomy [89]. Another study found that ultrasound transversus abdominal plane (US-TAP) wound infiltration combined with ultrasound transversus abdominal block is safe and effective for patients undergoing robotic prostatectomy and pelvic lymph node dissection [90].

Limitations of the evidence base

Despite covering UGRA, this review has limitations. As a narrative review, it lacks quantitative synthesis and a risk-of-bias assessment, thereby limiting causal inference. Though using six databases, publication and selection bias cannot be ruled out due to reliance on author judgment. Only English studies from January 2015 to October 2025 were included, potentially excluding relevant non-English or out-of-date research, thereby increasing selection bias. The review aimed to cover different clinical settings, but variability in study design, agents, and techniques affects comparability. Many studies were single-center, small, or observational and did not fully reflect real-world variability. Particularly for new technologies such as AI and robotics, there is a dearth of research on long-term outcomes, including recovery and chronic pain. Rapid tech evolution may render some conclusions outdated; future well-designed systematic reviews are needed. Furthermore, human trials involving AI, 3D/ultra-high-frequency ultrasound, Doppler technology, augmented reality, and robotics are scarce. Existing studies mainly involve cadavers or lack sufficient sample sizes to validate accuracy. The available evidence is limited by design variability, inconsistent reporting of outcomes, small sample sizes, and a focus on observational studies, all of which hinder direct comparisons and definitive conclusions.

## Conclusions

UGRA has emerged as an essential component of modern perioperative management, maximizing precision, safety, and analgesic efficacy across a variety of clinical settings. This narrative review focuses on the clinical applications, pharmacological considerations, and safety issues of modern UGRA, emphasizing its role in reducing opioid consumption and improving perioperative recovery profiles. Although evidence endorses its clinical utility, variability in techniques complicates direct comparisons among interventions. Advances in ultrasound technology, education, and supplementary techniques will continue to improve clinical outcomes in UGRA. Future studies should emphasize standardized outcome measures and comparative effectiveness studies to define best practices in UGRA.
